# Association between lipoprotein cholesterol and future cardiovascular disease and mortality in older adults: a Korean nationwide longitudinal study

**DOI:** 10.1186/s12944-020-01426-0

**Published:** 2021-01-06

**Authors:** Seung Hee Kim, Ki Young Son

**Affiliations:** Department of Family Medicine, Asan Medical Center, University of Ulsan College of Medicine, 88 Olympic-ro 43 gil, Songpa-gu, 05505 Seoul, Republic of Korea

**Keywords:** Lipoprotein cholesterol, Cardiovascular disease, Relationship, Elderly, Mortality, Incidence, Risk factor

## Abstract

**Background:**

Dyslipidemia is considered an independent health risk factor of cardiovascular disease (CVD), a leading cause of mortality in older adults. Despite its importance, there have been few reports on the association between lipoprotein cholesterol and future CVD and cardiovascular (CV) mortality among elderly Asians aged ≥ 65 years. This study investigated the association between lipoprotein cholesterol and future CVD and CV mortality in an elderly Korean population using a large nationwide sample.

**Methods:**

From the cohort database of the Korean National Health Insurance Service, 62,604 adults aged ≥ 65 years (32,584 men and 30,020 women) were included. High-density lipoprotein cholesterol (HDL-C) and low-density lipoprotein cholesterol (LDL-C) levels were categorized by quartiles. Cox proportional hazard models and linear regression analyses were used to assess the association between the quartiles of lipoprotein cholesterol and future CV events or mortality.

**Results:**

The mean follow-up period was 3.3 years. The incidence rates of ischemic heart disease and ischemic brain disease were 0.97 and 0.61 per 1,000 person-years, respectively, and the mortality rates from these diseases were 0.22 and 0.34 per 1,000 person-years, respectively. In a completely adjusted model, high HDL-C and LDL-C levels were not associated with total CV events and CVD mortality. However, high LDL-C levels were significantly associated with a lower incidence of ischemic brain disease. Furthermore, diabetic patients with high LDL-C levels were more likely to have higher CV mortality, whereas non-smokers with high LDL-C levels were less likely to be at risk of CV events.

**Conclusions:**

Neither high LDL-C nor HDL-C levels were significantly associated with future CV mortality in older adults aged ≥ 65 years. High LDL-C levels do not seem to be a risk factor for CVD in elderly individuals, and further studies are required.

## Background

Cardiovascular disease (CVD) is the most common cause of death globally [1]. According to the recently published data by the Organisation for Economic Co-operation and Development (OECD) Health Statistics 2020, the overall cardiovascular (CV) mortality rate for OECD member countries was 274.2 per 100,000 people, which was significantly higher than that for cancer [2]. Dyslipidemia is considered an independent risk factor for CVD. Low-density lipoprotein cholesterol (LDL-C) has been reported as the most atherogenic lipoprotein, and an interventional study has shown that lowering LDL-C levels using statin therapy reduces CV events [3]. All CV guidelines highlight the evidence that LDL-C is a major cause of CVD and a primary target of lipid-lowering therapy [4]. However, increase in high-density lipoprotein cholesterol (HDL-C) levels is not always associated with a positive effect, and the effect of lowering HDL-C levels is unclear [3].

As the elderly population has increased worldwide [5], CVD and its risk factors are important health problems for elderly individuals. Although CVD is a leading cause of mortality in older adults [6], only a few studies have assessed the associations between lipoprotein cholesterol and future CVD incidence or CV mortality in older populations. In some studies involving older adults, low HDL-C and high LDL-C levels were associated with an increase in CV events or mortality rates [7, 8]. In other studies, a low HDL-C level was significantly associated with the risk of stroke or CV mortality in elderly individuals, regardless of the LDL-C level [9, 10]. However, recent studies have shown no or an inverse association between LDL-C and mortality among older adults [11–14].

Moreover, those studies were limited to specific populations or a small number of participants, and even within the elderly population, the age range of the participants varied. There are relatively few studies assessing the association between lipoprotein cholesterol and future CVD and CV mortality in older Asian adults. In a Taiwanese study, elderly women with low total cholesterol, low LDL-C, or low HDL-C levels had higher CV mortality [15]; however, these results were inconsistent with those of a recent American study that showed that LDL-C was not associated with CVD risk in adults aged ≥ 75 years [16]. Therefore, more Asian studies are required to confirm the association between lipoprotein cholesterol and future CVD and CV mortality in the elderly population. This study aimed to evaluate these associations in individuals aged ≥ 65 years without a past medical history of dyslipidemia and CVD using a nationwide representative sample of elderly Korean individuals.

## Methods

The Korean National Health Service provides universal health insurance to almost all Koreans, except approximately 2.8% medical aid beneficiaries. The Korean National Health Insurance Service (KNHIS) provides the National Screening Program (NSP) biennially. The KNHIS established the National Health Information Database, including data from the results of NSP and variables related to sociodemographic characteristics and Korean mortality [17].

### Data sources and study population

**Fig. 1:**
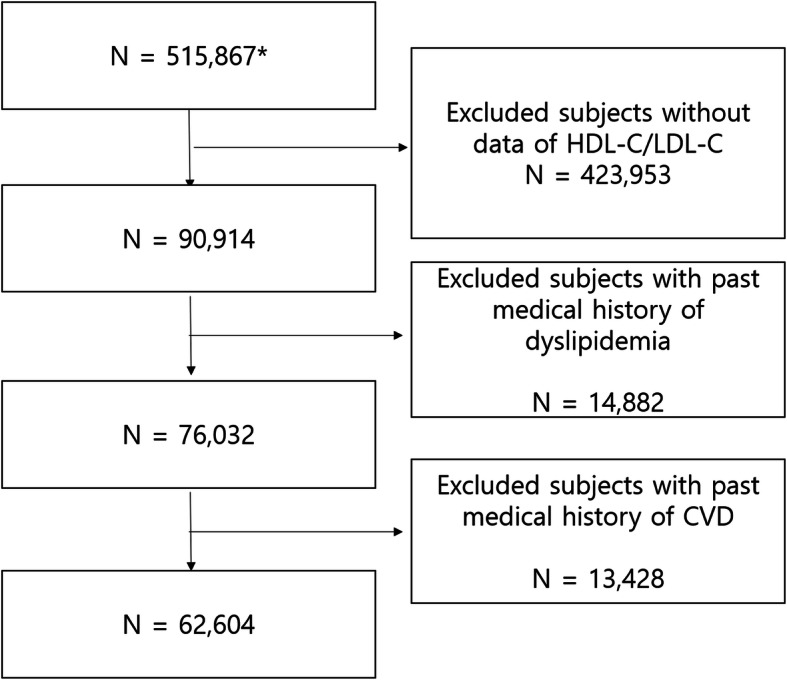
Participant selection. *Number of subjects in original the National Health Insurance Service-National Health Screening Cohort (2002–2015). **Abbreviation NSPTA: National Screening Program in Transitional Age, KNDR: Korean National Disability Registry, ADL: Activity of Daily Living

In the National Health Insurance Service–National Health Screening Cohort database of the KNHIS, 515,867 participants were included. The participants were randomly selected from health screening participants aged 40–79 years between 2002 and 2003. After excluding participants without data on HDL-C and LDL-C levels, those aged < 65 years, those who had a medical history of dyslipidemia (including those who had taken dyslipidemia medications), and those with a past medical history of CVD, 62,604 participants were included in this study (Fig. 1).

This study protocol was approved by the Institutional Review Board (IRB) of Asan Medical Center (IRB No. 2020 − 0649), and the need for informed consent was waived.

### Variables

#### Independent variable

##### Lipoprotein cholesterol

Lipids were assayed using an 8-hour fasting serum sample of participants in each community hospital. HDL-C was obtained as a measured value. LDL-C was calculated in samples with triglyceride levels < 400 mg/dL using the Friedewald formula [18], and it was obtained as a measured value in samples with triglyceride levels ≥ 400 mg/dL, similarly as a previous Korean study on LDL-C [19]. HDL-C and LDL-C values were categorized by quartiles.

#### Outcome variables

##### CV events and deaths from CVD

The International Classification of Diseases 10th Revision codes were reviewed for the classification of diagnosis and causes of death [20]. CVD was defined as ischemic heart disease (I20-25) and ischemic brain disease (I63), and CV events or deaths from CVD were defined as diagnoses of or deaths caused by ischemic heart diseases or ischemic brain diseases, including angina pectoris, acute or subsequent myocardial infarction, certain current complications following acute myocardial infarction, other acute or chronic ischemic heart diseases, and cerebral infarction.

Data regarding the diagnosis and date of CVD event as well as the cause and date of death were collected from the KNHIS cohort database during 2007–2015. As all participants were enrollees of the National Health Insurance or medical aid beneficiaries in Korea, dropout was almost impossible for reasons other than death.

The follow-up time for CV event was calculated as the time from the day of health examination in the NSP to the day of first diagnosis of CVD or December 31, 2015 for participants without events. The observation time for CV mortality was, therefore, defined as the time from the day of medical examination in the NSP to the day of death from CVD or the end of 2015 for participants without a reported date of death.

### Potential confounders

Age, sex, cigarette smoking status, and body mass index (BMI) were evaluated. Concerning the cigarette smoking status, participants were classified as non-smokers, ex-smokers, or current smokers. Non-smokers were defined as adults who had not smoked at least 100 cigarettes in their lifetime, ex-smokers were defined as adults who had smoked at least 100 cigarettes but were not currently smoking, and current smokers were defined as adults who had smoked at least 100 cigarettes and were currently smoking. BMI was calculated as body weight divided by the square of the height (kg/m^2^). According to the definition of obesity for Asians [21], BMI was categorized into normal (BMI < 23 kg/m^2^), overweight (BMI 23–25 kg/m^2^), and obese (BMI ≥ 25 kg/m^2^). Data on hypertension and diabetes were collected using a questionnaire. If participants reported using medications for hypertension or diabetes, they were considered to have the diseases. Hypertension was defined as systolic blood pressure ≥ 140 mmHg or diastolic blood pressure ≥ 90 mmHg. Diabetes was defined as fasting serum glucose levels ≥ 126 mg/dL. Each chronic disease was defined as described previously [22].

### Statistical analyses

For baseline characteristics, continuous variables were presented as mean ± standard deviation, and categorical variables were presented as frequencies and percentages.

Proportions of CV events and deaths were calculated according to the quartiles of lipoprotein cholesterol. Cox proportional hazard models and linear regression analyses were used to investigate the association between the quartiles of lipoprotein cholesterol and CV events or mortality. Four models were constructed for analyses—a crude model and three adjusted models. Model 1 was adjusted for age and sex; Model 2 was additionally adjusted for cigarette smoking status; and Model 3 was additionally adjusted for hypertension, diabetes, and BMI. In the Cox proportional hazard model, participants who did not have CV events but died before December 31, 2015 were right censored. Hazard ratios (HRs) and 95% confidence intervals (CIs) were obtained using Cox proportional hazard models, and beta coefficient (β) and *P*-values were calculated using linear regression analyses. To exclude reverse causality, an additional analysis was performed in Model 3, excluding elderly individuals who died within 1 year from the time of study.

Participants at a risk of future CVD events or CV death were assessed by stratified analyses in Model 3, which was stratified by sex, cigarette smoking status, and presence of hypertension, diabetes, and obesity. The purpose of this stratified analysis was to explore any effect modifier (interaction).

Statistical analyses were performed using STATA software (version 16.1; StataCorp, College Station, Texas). A *P*-value of < 0.05 was considered significant.

## Results

### Baseline characteristics of participants

Of the 62,604 participants included, 32,584 were men and 30,020 were women. There were 323 CV events and 114 CV deaths during the mean follow-up period of 3.3 ± 2.1 years (maximum: 9.0 years). The mean LDL-C values from the first, second, third, and fourth quartiles were 73.2 ± 15.1 mg/dL, 103.5 ± 6.5 mg/dL, 125.6 ± 6.9 mg/dL, and 161.6 ± 25.0 mg/dL, respectively. The mean HDL-C values from the first, second, third, and fourth quartiles were 38.7 ± 4.5 mg/dL, 48.5 ± 2.3 mg/dL, 56.7 ± 2.6 mg/dL, and 73.8 ± 30.2 mg/dL, respectively. The mean BMI of the participants was 24.1 ± 3.0 kg/m^2^, with 22,633 (36.2%) participants classified under the obese category (BMI ≥ 25 kg/m^2^). Of the total participants, 11.9% were current smokers, and 59.2% and 17.9% had been diagnosed with hypertension and diabetes, respectively. Each basic characteristic of the participants had a significant sex difference (Table 1).

**Table 1:** Basic characteristics of participants

	Total	Men	Women	*P*-value**
	*N* = 62604 (%)	*N* = 32584 (%)	*N* = 30020 (%)	
BMI (kg/m^2^)	24.1 ± 3.0	23.9 ± 2.8	24.4 ± 3.1	
<23	22395 (35.8)	12090 (37.1)	10305 (34.3)	< 0.001
23–25	17576 (28.1)	9543 (29.3)	8033 (26.8)	
≥25	22633 (36.2)	10957 (33.6)	11682 (38.9)	
Cigarette smoking				
Non-smoker	42630 (68.1)	13161 (40,4)	29469 (98.2)	< 0.001
Ex-smoker	12510 (20.0)	12331 (37.9)	179 (0.6)	
Current smoker	7439 (11.9)	7072 (21.7)	367 (1.2)	
LDL-C (mg/dL)	117.8 ± 35.3	112.0 ± 33.5	124.1 ± 36.1	
1st quartile	73.2 ± 15.1	72.4 ± 15.6	74.5 ± 14.1	< 0.001
2nd quartile	103.5 ± 6.5	103.3 ± 6.6	103.7 ± 6.5	
3rd quartile	125.6 ± 6.9	125.6 ± 6.9	126.3 ± 6.8	
4th quartile	161.6 ± 25.0	159.3 ± 21.9	163.2 ± 26.8	
HDL-C (mg/dL)	53.9 ± 19.9	52.3 ± 18.9	55.7 ± 20.8	
1st quartile	38.7 ± 4.5	38.4 ± 4.6	39.1 ± 4.3	< 0.001
2nd quartile	48.5 ± 2.3	48.4 ± 2.3	48.6 ± 2.3	
3rd quartile	56.7 ± 2.6	56.6 ± 2.6	56.8 ± 2.6	
4th quartile	73.8 ± 30.2	73.7 ± 29.8	73.9 ± 30.5	
Hypertension	29755 (59.2)	15568 (59.6)	14187 (58.7)	0.049
Diabetes mellitus	11205 (17.9)	6665 (20.5)	4540 (15.1)	< 0.001

^*^Abbreviation: *BMI* Body mass index, *LDL-C* Low-density lipoprotein cholesterol, *HDL-C* High-density lipoprotein cholesterol

**P-value is for comparison between men and women

### CV events and CV mortality

During the observation period of 204,025.6 person-years, the incidence rates of ischemic heart disease and ischemic brain disease were 0.97 and 0.61 per 1,000 person-years, respectively. The mortality rates from these diseases were 0.22 and 0.34 per 1,000 person-years, respectively, during the observation period of 204,058.7 person-years (Table 2).

**Table 2:** Association between quartiles of low-density lipoprotein cholesterol and cardiovascular disease

	Event	Duration (PYs)	Incidence rate	Crude	Model 1	Model 2	Model 3
				HR (95% CI)	HR (95% CI)	HR (95% CI)	HR (95% CI)
				β x 10^4^ (*P*-value**)	β x 10^4^ (*P*-value**)	β x 10^4^ (*P*-value**)	β x 10^4^ (*P*-value**)
Cardiovascular event							
Ischemic heart disease	198	204025.6	0.970	0.90 (0.79–1.03)	0.90 (0.79–1.03)	0.95 (0.83–1.09)	0.95 (0.83–1.09)
				-4.170 (0.041)	-2.874 (0.164)	-2.874 (0.165)	-1.159 (0.633)
Ischemic brain disease	125	204025.6	0.613	0.80 (0.68–0.94)	0.83 (0.70–0.98)	0.83 (0.70–0.98)	0.84 (0.70–1.00)
				-4.019 (0.013)	-3.450 (0.036)	-3.454 (0.036)	-3.389 (0.078)
Total	323	204025.6	1.583	0.83 (0.75–0.92)	0.87 (0.79–0.97)	0.90 (0.81–1.01)	0.90 (0.81–1.01)
				-8.189 (0.002)	-6.324 (0.017)	-6.328 (0.016)	-4.548 (0.142)
Cardiovascular death							
Ischemic heart disease	45	204058.7	0.221	0.88 (0.68–1.16)	0.99 (0.75–1.29)	0.99 (0.75–1.29)	1.13 (0.83–1.54)
				-0.708 (0.467)	-0.017 (0.986)	-0.022 (0.982)	0.961 (0.381)
Ischemic brain disease	69	204058.7	0.338	0.90 (0.73–1.12)	0.93 (0.75–1.16)	0.93 (0.75–1.16)	0.95 (0.74–1.22)
				0.572 (0.543)	0.934 (0.327)	0.932 (0.329)	1.709 (0.103)
Total	114	204058.7	0.559	0.89 (0.76–1.06)	0.95 (0.80–1.13)	0.95 (0.80–1.13)	1.01 (0.84–1.23)
				-1.617 (0.297)	-0.655 (0.677)	-0.663 (0.673)	-0.701 (0.687)

Model 1: age, sex

Model 2: Model 1 + cigarette smoking

Model 3: Model 2 + hypertension, diabetes mellitus, body mass index

^*^Abbreviation: *PY* Person-year, *HR* Hazard ratio, *CI* Confidence interval

Incidence rate is for the total participants.

HR is for the 4th quartile compared to the 1st quartile.

** *P*-value is for β coefficient of multiple linear regression analysis.

### Association between quartiles of LDL-C and CVD

**Fig. 2:**
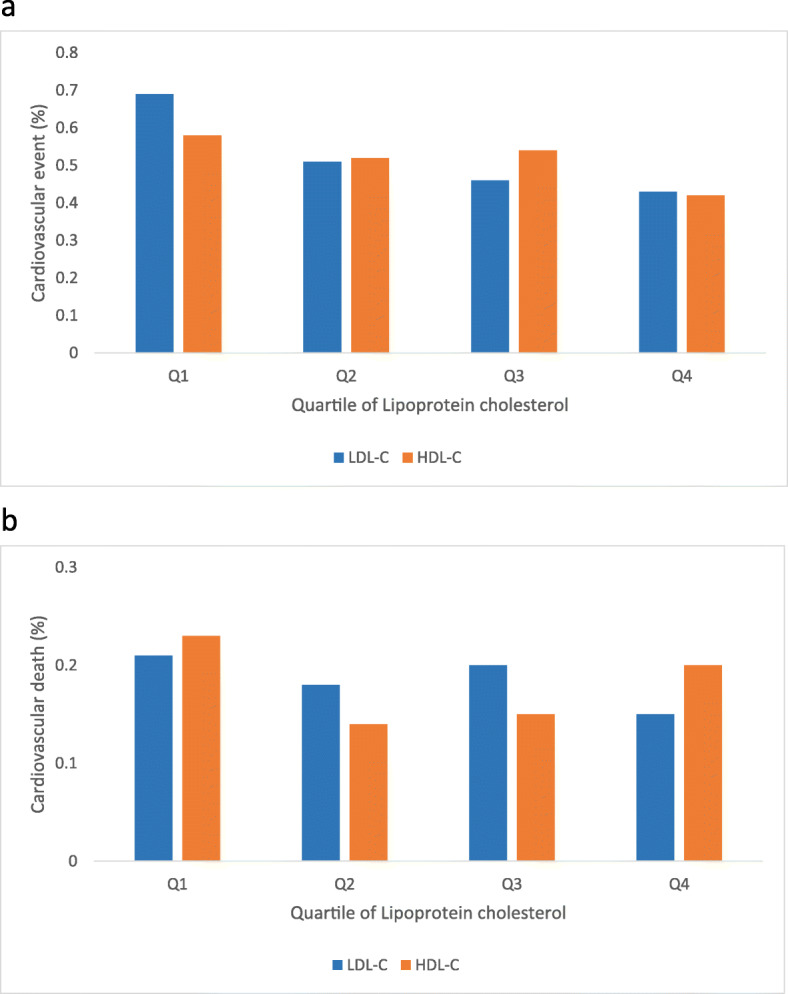
Proportions of cardiovascular events and deaths according to quartiles of lipoprotein cholesterol. **a** Proportion of cardiovascular events, **b** Proportion of cardiovascular deaths. ^*^Abbreviations: LDL-C, low-density lipoprotein cholesterol; HDL-C, high-density lipoprotein cholesterol

Proportions of CV events and deaths according to the quartiles of LDL-C are presented in Fig. 2. The proportion of CV events was the highest in participants with LDL-C values from the first quartile, and it decreased as the quartile of LDL-C increased. However, the CV death rate showed no specific pattern in accordance with the quartiles of LDL-C.

In the crude model (Table 2), the incidence of ischemic brain disease was 20% lower in participants with LDL-C values from the fourth quartile (HR: 0.80, 95% CI: 0.68–0.94) than in those with LDL-C values from the first quartile. However, mortality from ischemic brain disease was not significantly related to LDL-C levels (HR: 0.90, 95% CI: 0.73–1.12). Ischemic heart disease events and deaths were not significantly associated with high LDL-C levels. The total CV events were 17% lower in participants with LDL-C values from the fourth quartile (HR: 0.83, 95% CI: 0.75–0.92) than in those with LDL-C values from the first quartile. However, there was no significant association between total CV mortality and high LDL-C levels (HR: 0.89, 95% CI: 0.76–1.06). The incidences of ischemic heart disease (β = -4.17 × 10^− 4^, *P* = 0.041), ischemic brain disease (β = -4.02 × 10^− 4^, *P* = 0.013), and total CV events (β = -8.19 × 10^− 4^, *P* = 0.002) had negative correlations with LDL-C levels in the crude model by linear regression analysis.

In Models 1 and 2, the incidence of ischemic brain disease was 17% lower in participants with LDL-C values from the fourth quartile (HR: 0.83, 95% CI: 0.70–0.98 in both models) than in those with LDL-C values from the first quartile. However, mortality from ischemic brain disease was not significantly associated with LDL-C levels (HR: 0.93, 95% CI: 0.75–1.16 in both models). Ischemic heart disease events and mortality were not significantly related to high LDL-C levels. In Model 1, total CV events were 13% lower in participants with LDL-C values from the fourth quartile (HR: 0.87, 95% CI: 0.79–0.97) than in those with LDL-C value from the first quartile, but the relationship between them was not significant in Model 2 (HR: 0.90, 95% CI: 0.81–1.01). Total CV mortality was not associated with high LDL-C levels (HR: 0.95, 95% CI: 0.80–1.13 in both models). The incidences of ischemic brain disease and total CV events had negative correlations with LDL-C levels in Models 1 and 2 by linear regression analyses.

In Model 3, the incidence of ischemic brain disease was 16% lower in participants with LDL-C value from the fourth quartile (HR: 0.84, 95% CI: 0.70–1.00) than in those with LDL-C valued from the first quartile. However, mortality from ischemic brain disease was not significantly associated with LDL-C levels (HR: 0.95, 95% CI: 0.74–1.22). There was no significant association between the incidence of ischemic heart disease events or mortality and high LDL-C levels, and there was no association between total CV events or mortality and high LDL-C levels. In total, 202 participants died and CV deaths accounted for 22 cases within 1 year from the study. The results from the analysis of Model 3, excluding elderly individuals who died within 1 year from the time of study, were not different; total CV events and CV deaths were not significantly associated with LDL-C levels.

### Association between quartiles of HDL-C and CVD

Proportions of CV events and deaths according to quartiles of HDL-C are presented in Fig. 2. The proportion of CV events was the lowest in participants with HDL-C valued from the fourth quartile. An U-shaped association was observed between the proportion of CV deaths and HDL-C levels.

In the crude model (Table 3), ischemic heart disease events were 12% lower in participants with HDL-C values from the fourth quartile (HR: 0.88, 95% CI: 0.77–1.00) than in those with HDL-C values from the first quartile. However, mortality from ischemic heart disease was not significantly lowered (HR: 0.79, 95% CI: 0.61–1.04). Ischemic brain disease events (HR: 0.95, 95% CI: 0.82–1.12) and deaths (HR: 1.04, 95% CI: 0.84–1.28) were not significantly associated with high HDL-C levels. Total CV events were 9% lower in participants with HDL-C values from the fourth quartile (HR: 0.91, 95% CI: 0.82–1.00) than in those with HDL-C values from the first quartile. However, there was no significant association between total CV mortality and high HDL-C levels (HR: 0.93, 95% CI: 0.79–1.10). The incidence of total CV events had a negative correlation with HDL-C levels (β = -4.57 × 10^− 4^, *P* = 0.041) in the crude model by linear regression analysis.

**Table 3:** Association between quartiles of high-density lipoprotein cholesterol and cardiovascular disease

	Event	Duration (PYs)	Incidence rate	Crude	Model 1	Model 2	Model 3
				HR (95% CI)	HR (95% CI)	HR (95% CI)	HR (95% CI)
				β x 10^4^ (*P*-value**)	β x 10^4^ (*P*-value**)	β x 10^4^ (*P*-value**)	β x 10^4^ (*P*-value**)
Cardiovascular event							
Ischemic heart disease	198	204025.6	0.970	0.88 (0.77–1.00)	0.90 (0.80–1.03)	0.90 (0.80–1.03)	0.95 (0.83–1.09)
				-3.789 (0.057)	-2.804 (0.163)	-2.832 (0.159)	-1.293 (0.585)
Ischemic brain disease	125	204025.6	0.613	0.95 (0.82–1.12)	0.98 (0.83–1.14)	0.98 (0.84–1.15)	0.94 (0.79–1.12)
				-0.784 (0.621)	-0.292 (0.855)	-0.262 (0.870)	-0.960 (0.610)
Total	323	204025.6	1.583	0.91 (0.82–1.00)	0.93 (0.85–1.03)	0.93 (0.85–1.03)	0.95 (0.85–1.05)
				-4.573 (0.041)	-3.096 (0.164)	-3.094 (0.165)	-2.252 (0.633)
Cardiovascular death							
Ischemic heart disease	45	204058.7	0.221	0.79 (0.61–1.04)	0.84 (0.65–1.10)	0.85 (0.65–1.11)	0.82 (0.60–1.11)
				-1.543 (0.105)	-1.041 (0.278)	-0.993 (0.301)	-1.237 (0.248)
Ischemic brain disease	69	204058.7	0.338	1.04 (0.84–1.28)	1.06 (0.86–1.31)	1.06 (0.86–1.31)	1.10 (0.87–1.40)
				-1.121 (0.223)	-0.881 (0.342)	-0.846 (0.361)	-0.551 (0.591)
Total	114	204058.7	0.559	0.93 (0.79–1.10)	0.98 (0.83–1.15)	0.98 (0.83–1.15)	0.98 (0.81–1.19)
				-1.043 (0.491)	-0.312 (0.838)	-0.222 (0.884)	-0.113 (0.947)

Model 1: age, sex

Model 2: Model 1 + cigarette smoking

Model 3: Model 2 + hypertension, diabetes mellitus, body mass index

^*^Abbreviation: *PY* Person-year, *HR* Hazard ratio, *CI* Confidence interval

Incidence rate is for the total participants.

HR is for the 4th quartile compared to the 1st quartile.

** *P*-value is for β coefficient of multiple linear regression analysis.

In Models 1, 2, and 3, the incidence of CV events and CV mortality were not significantly associated with high HDL-C levels. The result from the analysis of Model 3, excluding elderly individuals who died within 1 year from the time of study, was not different; total CV events and CV deaths were not significantly associated with HDL-C levels.

### Stratified analysis

**Fig. 3:**
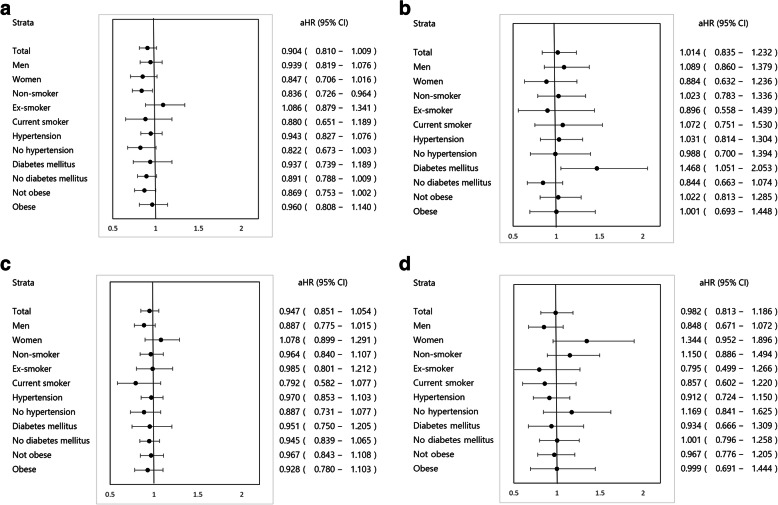
Forest plots showing stratified analyses of the association between lipoprotein cholesterol and cardiovascular events and mortality. **a** LDL-C and cardiovascular event, **b** LDL-C and cardiovascular death, **c** HDL-C and cardiovascular event, **d** HDL-C and cardiovascular death. ^*^Abbreviations: aHR, adjusted hazard ratio; CI, confidence interval; LDL-C, low-density lipoprotein cholesterol; HDL-C, high-density lipoprotein cholesterol. aHR is for the fourth quartile compared to the first quartile

In a stratified analysis of the association between LDL-C levels and CV events, the risk of CV events in non-smokers with LDL-C values from the fourth quartile was significantly reduced (adjusted HR [aHR]: 0.84, 95% CI: 0.73–0.96) than in those with LDL-C values from the first quartile (Fig. 3a). In a stratified analysis of the association between LDL-C levels and CV deaths, the CV mortality rate in diabetic patients with LDL-C values from the fourth quartile increased significantly (aHR: 1.47, 95% CI: 1.05–2.05) than in those with LDL-C values from the first quartile (Fig. 3b). Stratified analysis could not find any strata with a significant association between HDL-C levels and CV events or deaths (Fig. 3c, d).

## Discussion

This study examined the association between lipoprotein cholesterol and future CVD events and deaths using a nationwide large sample of an Asian country. In the completely adjusted model, HDL-C levels were not associated with the incidences of CVD or CV mortality. However, high LDL-C levels were significantly associated with a lower incidence of ischemic brain disease, although they were not associated with ischemic heart disease events or CV death. In stratified analyses, diabetic patients with high LDL-C levels had a higher CV mortality rate than those with low LDL-C levels, and non-smokers with high LDL-C levels had a lower risk of CV events than those with low LDL-C levels, showing interactions between LDL-C levels, diabetes, and smoking.

There was no significant association between LDL-C levels and CV mortality, which was robust in stratified analyses, consistent with the results of other studies in older adults [10, 11, 23, 24]. However, this finding contradicts the findings from studies in younger adults, in which high LDL-C and low HDL-C levels were associated with an increased risk of CV mortality [25]. This inconsistency may be due to biological differences according to the age and age-related confounding factors [26]. One of the possible reasons why studies in younger people have shown an association between LDL-C levels and CVD is that younger people are more stressed than retired older people, and stress may increase cholesterol levels by up to 40% [27]. Moreover, other mechanisms that increase cholesterol levels may cause CVD [28]. Cholesterol levels tend to decrease with age [29], suggesting that the role of cholesterol in determining the risk of CVD may become less relevant in a more aged population [23]. In addition, HDL-C levels were not associated with the incidence of CVD and CV mortality in this study, which is consistent with the findings of previous studies [11, 30]. These results differ from those of other studies in which increased HDL-C levels were associated with a reduced risk of CVD in elderly individuals [31], presumably because the effect of other variables affecting CVD was greater than that of HDL-C in this study. The association between HDL-C levels and CV events was attenuated after adjusting for CV risk factors, which suggests that the observed association could be due to residual confounding factors, as reported in a prior study [11].

The findings of this nationwide longitudinal study including a large Korean elderly population aged ≥ 65 years reinforces the findings of most previous studies involving elderly individuals that high of LDL-C and HDL-C levels were not significantly associated with total CV events and CV mortality. Furthermore, this finding is consistent with that of a previous study suggesting that lowering LDL-C levels using statin therapy was not effective in the primary prevention of CV events or deaths in older adults aged ≥ 70 years [32].

High LDL-C levels were significantly associated with a lower incidence of ischemic brain disease in older adults, which contradicts the hypothesis that cholesterol, particularly LDL-C, is inherently atherogenic. A systematic review demonstrated that elevated LDL-C levels were inversely associated with all-cause mortality, and CV mortality was significantly higher in the lowest LDL-C quartile in older adults [12]. One of the possible reasons for these findings is that elevated cholesterol levels could be protective in weak older survivors. As cholesterol has various physiological functions, including nerve conduction and intracellular transport, and is a part of all cell membranes and a precursor for the synthesis of substances vital for the organism [33], elevated LDL-C levels may have played a role in protecting frail individuals and those with other catabolic states. Another possible explanation is that healthy elderly survivors may be less susceptible to the negative effects of high LDL-C levels [34], and higher LDL-C levels are likely to be significantly associated with healthy survival, as reported in a recent study [11]. One study suggested that low total cholesterol levels may be a biomarker for malnutrition-related illness in older persons [35], while other studies have reported that higher cholesterol levels were associated with better outcomes in late-life physical function and the ability to recover from illnesses [36, 37]. Another possible reason of the inverse association between CVD and LDL-C levels is that CVD may be caused by infections, and high LDL-C levels may be beneficial as LDL is involved in the immune system by adhering to and inactivating all kinds of microorganisms and their toxic products [38, 39]. Moreover, the mean LDL-C value in the fourth quartile in this study was 161.6 ± 25.0 mg/dL, which was relatively lower than that in the highest quartile in most studies showing no association between high LDL-C levels and CV mortality [10, 11, 23, 26, 40] because older adults who had a medical history of dyslipidemia (including those who had received medications for dyslipidemia) were excluded from this study. It seems that LDL-C levels about 160 mg/dL had a protective effect in older people, reducing the incidence of ischemic brain disease.

Although the risk of CVD death was not significantly associated with high LDL-C levels in older adults without diabetes, it was significantly increased in diabetic patients with high LDL-C levels, showing an interaction between LDL-C levels and diabetes. Older adults with diabetes are particularly at a high risk of CVD mortality. Elderly patients with diabetes may have less end-organ reserve due to aging and comorbidities, which could result in more abrupt and severe CVD [41]. In a previous study, increased LDL-C levels were associated with an increased risk of CVD mortality in individuals with type 2 diabetes [42]. However, most previous studies have shown that LDL-C is not associated with mortality among diabetic patients [43–46], and a systematic review has shown that dyslipidemia treatment is unable to prevent CVD in patients with diabetes [47]. These suggest that including dyslipidemia treatment in patients with diabetes is not beneficial in preventing CVD death and that diabetes, not high LDL-C levels, is a major predictor of CV mortality in patients with diabetes.

Non-smokers with high LDL-C levels were had a significantly lower risk of CV events; however, the association between LDL-C and future CVD was not significant in ex-smokers and current smokers. Thus, there was an interaction between smoking and LDL-C. Smoking has been reported as an independent predictor of CV incidence in older and middle-aged population, and there was nearly a two-fold increase in its absolute risk in elderly individuals [48]. There was an inverse association between LDL-C and a risk of future CVD in non-smokers.

### Study strengths and limitations

This study has its strengths. This was a nationwide longitudinal study comprising a large elderly population. Moreover, in this study, the main CV risk factors were adjusted. Nevertheless, this study has several limitations. The mean follow-up time of 3.3 years was relatively shorter than that of other studies reporting future CV morbidity and mortality. It is possible that other confounding factors affecting CVD have not been considered. Furthermore, due to the exclusion of elderly patients with dyslipidemia to rule out the effect of statins, future CV incidence and mortality in older adults with higher LDL-C levels or those taking statins could not be identified. Considering that the participants were all elderly Korean individuals, it is difficult to generalize the results to other elderly populations. With the cohort effect that only relatively healthy older adults live up to a significantly old age, the results should be interpreted carefully considering survivor bias.

## Conclusions

Neither LDL-C nor HDL-C levels were significantly associated with future CV death in older adults aged ≥ 65 years, except high LDL-C level, which was significantly associated with a lower incidence of ischemic brain disease. In stratified analysis, high LDL-C levels were associated with an increased risk of CV mortality in older adults with diabetes. However, according to several previous studies, there was no association between high LDL-C levels and mortality in patients with diabetes and cholesterol-lowering trial could not reduce mortality in people with diabetes. Further studies are required to confirm whether lowering LDL-C levels in older adults with diabetes is beneficial.

## Data Availability

The dataset supporting the findings of this article are available from the National Health Insurance Sharing Service, but restrictions apply to the availability of these data, which were used under license for the current study. Hence, they are not publicly available. Data are, however, available on request. The information on how to request for database is provided at https://nhiss.nhis.or.kr/bd/ab/bdaba021eng.do. The database used for this study can be requested at https://nhiss.nhis.or.kr/bd/ay/bdaya001iv.do. The questionnaire used in this study is available for download at http://www.law.go.kr/admRulLsInfoP.do?chrClsCd=&admRulSeq=2200000012541#AJAX.

## Abbreviations

CVD: Cardiovascular disease
HDL-C: High-density lipoprotein cholesterol
LDL-C: Low-density lipoprotein cholesterol
CV: Cardiovascular
KNHIS: Korean National Health Insurance Service
NSP: National Screening Program
IRB: Institutional Review Board
BMI: Body mass index
HR: Hazard ratio
aHR: Adjusted hazard ratio
CI: Confidence interval
